# *Hydnum pallidum* Raddi, the Correct Name for *H. albidum* Peck in the Sense of European Authors and the Recently Described *H. reginae* Kibby, Liimat. & Niskanen

**DOI:** 10.3390/jof9121141

**Published:** 2023-11-25

**Authors:** Rodrigo Márquez-Sanz, Sergio Pérez Gorjón, Isabel Salcedo, Ibai Olariaga

**Affiliations:** 1 Biology and Geology, Physics and Inorganic Chemistry Department, Rey Juan Carlos University, C/Tulipán s/n, 28933 Móstoles, Spain; ibai.olariaga@urjc.es; 2 Department of Botany and Plant Physiology, Faculty of Biology, Plant DNA-Biobank, University of Salamanca. C/Licenciado Méndez Nieto s/n, 37007 Salamanca, Spain; spgorjon@usal.es; 3 Department of Plant Biology and Ecology (Botany), University of the Basque Country (UPV/EHU), Apdo 644, 48080 Bilbao, Spain; isabel.salcedo@ehu.eus; 4 Aranzadi Society of Sciences, Mycology Section, Zorroagagaina 11, 20014 Donostia-San Sebastián, Spain

**Keywords:** Cantharellales, *Hydnum album*, *Hydnum heimii*, species delimitation, red-listing

## Abstract

The systematics of the genus *Hydnum* have undergone important advances, and many new species have been described with the aid of molecular data. A revision of old names that refer to *Hydnum* s. str., considering the knowledge now available, might reveal prioritary names of recently described species. This study focuses on the study of names that refer to white *Hydnum* in Europe, among which earlier synonyms of *Hydnum reginae* (=*Hydnum albidum* s. auct. pl. eur.) are potentially found, a species characterized by producing white basidiomata and smaller spores than any other European species. Our revision revealed the existence of three earlier names based on European material, namely *H. pallidum* Raddi, *H. album* Fr. and *H. heimii* Maas Geest. The earliest of those, *Hydnum pallidum,* is epitypified using material from Tuscany (Italy), from where it was originally described, and hence, it becomes the correct name for *H. albidum* s. auct. pl. eur. A full description and photographs of *H. pallidum* are provided, and further comments on other names that refer to white *Hydnum* based on European material are made.

## 1. Introduction

*Hydnum* comprises ectomycorrhizal fungi that produce pileate and stipitate basidiomata with a spiny hymenophore, along with suburniform basidia of stichic type [1]. Its species are edible and popularly known as “hedgehogs” or “wood urchins”. For decades, only a handful of species were recognized in the world (in Europe, just *H. repandum* L., *H. rufescens* Pers. and *H. albidum* Peck), but the systematics of *Hydnum* have undergone important advances since molecular data, especially from the ITS region, became available [2,3,4], and currently 49 species are recognized in the world [5].

Although several new species of *Hydnum* were described as new recently [2,3,4], it remained evident for several years that some clades lacked a binomial name, and new names had to be coined for those. In this context, the contribution by Niskanen et al. [5] represented an important advance by naming the up-to-then known diversity of *Hydnum* based on the ITS region—twenty-two new species were described, along with epitypifying *Hydnum repandum* and *H. rufescens*. Since this study based the description of several species on a single or very few specimens, morphological characters could not always be revised in depth, and the morphological variations in those taxa are often poorly understood. In this context, species delimitation is far from being straightforward in *Hydnum* and largely relies on molecular data from the ITS region. Morphological characters that support species discrimination are often not clear-cut, namely basidioma robustness, pileus colour, the attachment of the spines disposition, along with the size and shape of basidiospores [5]. Old names that refer to *Hydnum* have been largely disregarded in recent studies, probably due to the fact that type specimens are lost or the difficulties in recognizing species morphologically. Maas Geesteranus [6] listed and studied in depth many names that need to be re-interpreted in this new context, as potentially prioritary older names could be available for newly described taxa.

This study focuses on the revision of old names that are applicable to what European mycologists have called *H. albidum*. Peck [7] described *H. albidum* from North America and stated that it differed from *H. repandum* in its smaller size, white colour and smaller spores. The first report of *H. albidum* in Europe was made by Maas Geesteranus [8] (p. 495), based also on the material of “a conspicuous whiteness, the spines very much crowded and more slender than in *H. repandum*, while its spores are smaller and narrower”. Subsequent authors followed Maas Geesteranus and applied the name *H. albidum* to European material with a white colour and small spores. Currently, reports attributed to *H. albidum* have been made from temperate and Mediterranean Europe, almost always in coniferous or broadleaf forests with calcareous ground: Austria [9], Denmark [10], France [11], Germany [12], Italy [13], Spain [14], Sweden [15], Switzerland [8,16], and the United Kingdom [17] (as *Hydnum reginae*). All the European material assigned to *H. albidum* appears to be homogeneous, and the species is considered easily recognized [18] and even striking in the field ([17] as *H. reginae*). Previous authors, Olariaga [18] (p. 326), adopted the name *H. albidum* but noted that the basidiospores of the Iberian material were ellipsoid—as opposed to the subglobose spores mentioned by Peck—and that several earlier putative synonyms described in Europe required further study. Swenie et al. [19] epitypified *H. albidum* after studying extensive material collected in the region of the type locality and providing sequences that considerably differ from the ones generated from European material identified as *H. albidum*. Once it became evident that *H. albidum* was being misapplied in Europe, Kibby and Liimatainen [17,20] erected a new species name, *H. reginae* Kibby, Liimat. & Niskanen, to accommodate collections previously referred to *H. albidum* in Europe. Being the only species of *Hydnum* sect. *Alba* Niskanen & Liimat. in Europe [5], the only other species that can be confused with *H. reginae* (=*H. albidum* s. auct. pl. eur.) is *H. boreorepandum* Niskanen, Liimat. & Niemelä, which differs in having larger spores, always-white spines and being only known from boreal forests in Finland [5] and Sweden (specimen S F-156605 recorded by Ibai Olariaga). *Hydnum vesterholtii* Olariaga, Grebenc, Salcedo & M.P. Martín and *H. ibericum* Olariaga, Liimat. & Niskanen also have a pale-coloured pileus but differ macroscopically in having a pale ochre colour, even in young basidiomata [3,5]. Thus, *H. albidum* s. auct. pl. eur. is a species that can be readily distinguished in the field in Europe, even more straightforwardly in the Mediterranean region.

A comprehensive nomenclatural revision, considering all currently available knowledge of *Hydnum*, revealed the existence of three names, *Hydnum pallidum* Raddi [21], *H. album* Fr. [22] and *H. heimii* Maas Geest. [6], that are synonyms and have priority over *H. reginae*. Thus, the goals of this study are to propose stable interpretations of those names and to contribute to nomenclatural stability before a name without priority, *H. reginae*, is widely adopted by the mycological community.

## 2. Materials and Methods

### 2.1. Morphological Studies

Specimens were studied from AGMT, AH, ARAN, BIO, C, GDAC, JA, MA, PI and S herbaria [23] and from the private herbaria of Marco Cartabia and Luis Ballester (LB). Thirty-five specimens, all of them previously identified as *H. albidum* Peck, were examined. Colour codes are based on the charts of The Royal Horticultural Society [24] and those of dried material on Munsell Color Corp. (Baltimore, MD, USA) [25]. Basidiospores were measured from dried material by rehydrating 1–3 spines in KOH 5%. Only released mature spores were measured. Spore statistics are based on measurements of 25 spores from each collection: Lm = mean length, Wm = mean width and Qm = Lm/Wm. The number of spores measured is provided as n = total number/number of specimens (e.g., n = 25/1). Extreme values are given in parentheses.

### 2.2. DNA Extraction, PCR Amplification, Sequencing and Alignment

DNA was extracted from dried material using either the DNeasy Plant Mini Kit (QIAGEN, Hilden, Germany) or the PTB DNA extraction protocol following [26]. The primer combinations used were ITS5-ITS4 [27] and LR0R-LR5 for the ITS and LSU [28], respectively. The same primers were employed for sequencing. PCRs were performed using a Master Mix (QIAGEN Multiplex PCR Kit) in a 20 µL volume and then conducted in an Applied Biosystems GeneAmp^®^ PCR System 9700 and 2720 Thermal Cyclers. The amplification programme used was initial denaturation at 95 °C for 5 min, followed by 35–45 cycles of 95 °C for 45–60 s, 50–55 °C for 50 s, 72 °C for 1 min, followed by a final extension at 72 °C for 10 min. PCR products were visualized in a 1% agarose gel, stained with SYBR Safe DNA Gel Stain (Invitrogen-Thermo Fisher Scientific, Inc., Waltham, MA, USA), and through a UV trans-illuminator. PCR products were sequenced at the Macrogen Spain service.

Sequences were edited and assembled using Sequencher v. 4.1.4 (Gene Codes Corp., Ann Arbor, MI, USA) and deposited in GenBank, and additional sequences were downloaded from the GenBank and UNITE databases (Table 1). The dataset was automatically aligned in Aliview v. 1.26 [29] and manually adjusted. The ITS-LSU data matrix was subjected to Maximum Likelihood (ML) and Bayesian analyses. The partitions of the alignment were inferred in ITSx [30] and subsequently analyzed in PartitionFinder v. 2.1.1 [31] using the greedy algorithm [32] in order to assess the best-fit evolution model for each region (ITS1, 5.8S, ITS2 and LSU).

### 2.3. Phylogenetic Analysis 

The ML analysis was conducted in IQ-TREE [33], starting from a random tree and setting four partitions (ITS1, 5.8S, ITS2, LSU) under default options. To assess the branch confidence, 1,000 ML bootstrap replicates were conducted using standard bootstrapping. The Bayesian analysis was carried out in MrBayes v. 3.2.7a [34], in the CIPRES Science Gateway [35], using two parallel runs of 4 Metropolis-coupled Markov chain Monte Carlo (MCMCMC) chains for 30 M generations, starting from a random tree, and sampling one tree every 1000 generations from the posterior distribution. Substitution models were sampled across the GTR space during the MCMC simulation [34]. Stationarity was assumed when the average standard deviation of split frequencies fell below 0.01. The burn-in fraction was set to discard 0.25 trees from each analysis. To assess the branch confidence, a 50% majority rule consensus tree was computed with the remaining trees using the SUMT command of MrBayes. An ML standard bootstrap (ML-Boot) ≥95 and Bayesian posterior probability (PP) values ≥ 0.95 were considered supported.

## 3. Results

### 3.1. Molecular Analysis

Eight new (six ITS and two LSU) sequences were generated in this study (Table 1). The ITS dataset comprised 49 sequences of 39 different taxa and a total of 1538 positions, of which 234 were parsimony informative and 600 distinct patterns. ITSx recovered four partitions, and the following best-fit evolution models were proposed by PartitionFinder: ITS1 (1-218, TRN + G), 5.8S (219-375, K80 + I + G), ITS2 (376-651, TIM + G) and LSU (652-1538, GTR + I + G).

**Figure 1:**
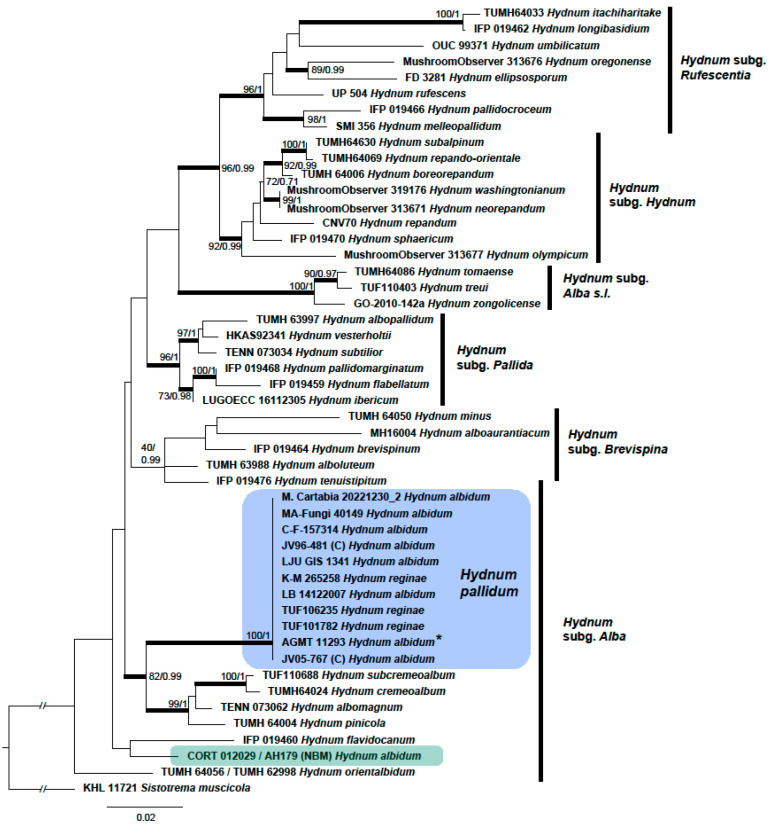
Best tree of the Maximum Likelihood analysis of selected sequences of *Hydnum* from ITS/LSU sequence data. Maximum Likelihood standard bootstrap values (ML-Boot)/Bayesian posterior probabilities (PP) are shown on branches, ordered as ML-UFBoot/PP. Thickened branches received support in both analyses (ML-Boot ≥ 95% and/or PP ≥ 0.95). Values are provided for nodes supported at least in one analysis. The epitype of *Hydnum pallidum* is marked by an asterisk. The *H. pallidum* clade is highlighted in a blue colour, whereas the American *H. albidum* is in green. Subgenera are indicated by vertical bars.

The ML analysis of the combined dataset resulted in a single best ML tree of -lnL = 6412.498. The Bayesian analyses reached an average standard deviation of split frequencies of 0.001699 after 30 M generations. A majority rule consensus tree was constructed from the 45,002 trees sampled from the two runs, each consisting of 22,501 trees sampled from the stationary tree distribution (the first 25% discarded as the burn-in) (Figure 1). The sequences of *H. pallidum*, including the two specimens from Tuscany, form a strongly supported clade (ML-Boot 100, BPP 1). *Sistotrema muscicola* (AJ606040, AJ606040) was set as an outgroup for the dataset, previously recovered as a sister clade to *Hydnum* within Cantharellales [36].

The sequences of *H. pallidum* (many of them identified as *H. albidum* or *H. reginae*; see Table 1) show a low sequence divergence and are identical except for the lack of a cytosine at position 142 of the alignment in LJU-GIS 1341 and some gaps at the start of the sequences TUF106235 and TUF101782, which might be due to sequencing errors.

### 3.2. Taxonomy

***Hydnum pallidum*** Raddi, Mem. Mat. Fis. Soc. Ital. Sci. Modena, Pt. Mem. Fis. 13(2): 353. 1807.

non *Hydnum pallidum* Cooke & Ellis, Grevillea 9: 103. 1881 (“1880–1881”, nom. illeg.). Lectotype of *Hydnum pallidum* Raddi (designated by Maas Geesteranus [6] (p. 135): Tab XIII, Figure 8). Epitype (here designated): Italy, Tuscany, under *Quercus ilex* with *Erica*, A. Cristiano, 26 December 2022, AGMT 11293. MycoBank MBT178088.

=*Hydnum album* Fr., Obs. Mycol. 1: 148. 1815. Lectotype (designated by Maas Geesteranus [6] (p. 15): Micheli, [37] Nova Pl. Gen.: Table 72, Figure 1729). Epitype (here designated): Italy, Tuscany, under *Quercus ilex* with *Erica*, A. Cristiano, 26 December 2022, AGMT 11293. MycoBank MBT178088

=*Hydnum heimii* Maas Geest., Persoonia 1: 133. 1959. (new name for *Sarcodon abietinus* R. Heim, [38] Rev. Mycol. (Paris) 8(1, supp.): 10. 1943.), as *Sarcodon abietinum*, nom. inval. Art. 39.1. Type: the specimens cited by Heim [38] are apparently lost, as noted by Maas Geesteranus [6] (p. 133). Lectotype: [39] Heim, Bull. Soc. Mycol. France 67(atlas): 99. 1952 (as *Sarcodon abietum*), indicated by Maas Geesteranus [40] (p. 27).

=*Hydnum reginae* Kibby, Liimat. & Niskanen, Index Fungorum 523: 1. 2022. Holotype: Great Britain, on soil, associated with Fagus, North Downs Way, White Downs, Surrey, 13 Oct. 2021, Coll. G. Kibby, M. Tortelli & C. Soler, K(M) 265258.

=*Sarcodon repandus* var. *albus* Quél., Fl. Mycol. France: 447. 1888. ≡ *Hydnum repandum* var. *album* (Quél.) Rea, Brit. Basidiomyc.: 630. 1922. ≡ *Dentinum repandum* var. *album* (Quél.) K.A. Harrison, Publ. Dept. Agric. Canada 1099: 19. 1961. Type: none. No original specimen appears to exist in PC herbarium.

—*Hydnum albidum* Peck, Bull. New York State Mus. Nat. Hist. 1(2): 10. 1887 [7], sensu auct. pl. eur. 

### 3.3. Description of Hydnum Pallidum

Pileus: 22–60 mm diam., fleshy, initially convex, plane afterward, sometimes depressed in the centre, often becoming irregular. Pileus surface slightly velutinous to smooth, staining, not zoned, cream white (158C, 158D, 159D) to pale ochre (159A), with yellow ochre (16D) zones or patches. Margin long involute, lobed in old basidiomata. In exsiccatum ochre (10YR 7/6, 8/6), sometimes with brownish orange (5YR 5/8) zones. Stipe 15–35 × 8–25 mm, tapering downwards, central or slightly excentric, solid, velutinous, cream white (158C, 158D, 159D), with yellow ochre (16D) patches. Staining: conspicuous, yellow ochre (16D). In exsiccatum ochre (10YR 7/6, 8/6), sometimes with brownish orange (5YR 5/8) zones. Spines: non-decurrent to slightly decurrent, conical, acute or obtuse, sometimes spathulate in old basidiomata, often fimbriate, sometimes joined at the base, crowded, 1.2–4 × 0.15–0.5 mm, initially cream white (158C, 158D), orangish ochre (18B, 23D) afterward. In exsiccatum, brownish ochre (10YR 5/8, 6/8). Context: cream white (158D), staining, faint smell and not distinctive, mild taste. Macrochemical reactions: KOH + context = nil; TL4 + context = nil; FeSO4 + context = nil. Spore print: pale orange ochre (158B).

**Figure 2:**
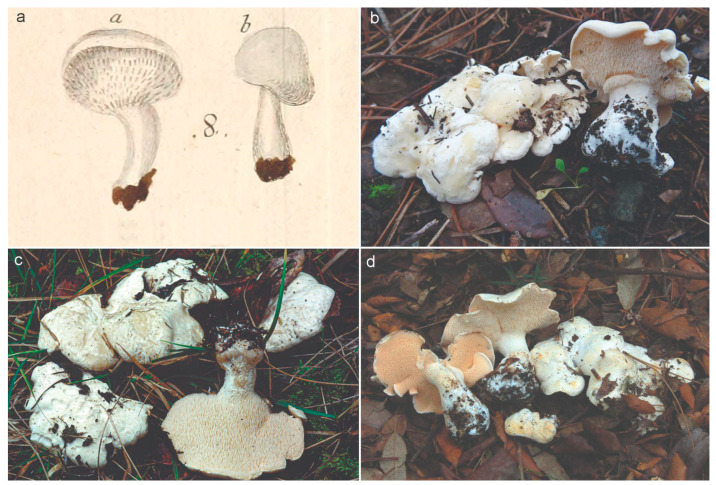
Type materials and photographs of fresh specimens of *Hydnum pallidum* for comparison. (**a**). Lectotype of *Hydnum pallidum* (Raddi, Mem. Mat. Fis. Soc. Ital. Sci. Modena, Pt. Mem. Fis. 13(2): Tab XIII, Figure 8. 1807). The a and b letters on the top of *H. pallidum* drawings belongs to original plate from Raddi. (**b**). Epitype specimen AGMT 11,293 from Tuscany (Italy). (**c**). Specimen JV96-401 (C-F-28274) from Tuscany (Italy). (**d**). Specimen BIO-Fungi 11,726 (Spain). Photographs Andrea Cristiano (**b**), Jan Vesterholt (**c**) and Ibai Olariaga (**d**).

**Figure 3:**
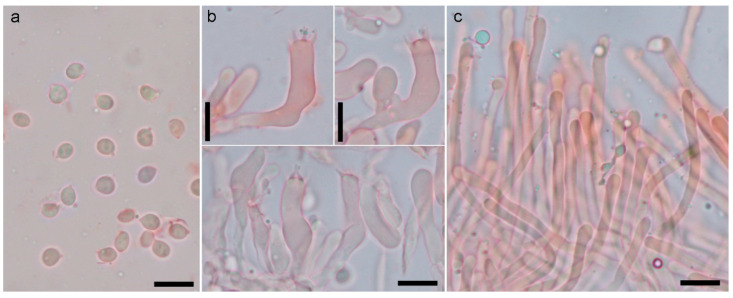
Microscopic characters of *Hydnum pallidum*, (epitype, AGMT 11293). (**a**). Basidiospores. (**b**). Basidia. (**c**). Hyphae from spine tips. Scale bars: 10 μm.

Basidiospores: broadly ellipsoidal to ellipsoidal in side-view, seldom partially cylindrical or amygdaliform, apiculus cubic, thin-walled, non-amyloid, 4.5–6.6 (–7.2) × 3.4–4.5 (–4.8) μm (Lm = 4.9–6.1, Wm = 3.7–4.2; Qm = 1.26–1.46; n = 350/14). Basidia: suburniform to claviform, 5–6-spored, sometimes with scattered 1-2-4-spored ones, clamped, 30–48 × 5–6.5 μm. Hyphae of the apex of the spines arranged parallel, cylindrical, thin-walled, light yellow, clamped, 2.5–5 μm, with cylindrical-to-claviform ends, often slightly to notably encrusted with rounded crystals (<1 μm). Pileipellis: composed of hyphae forming a cutis-trichoderm, cylindrical to swollen, thin-walled, hyaline to yellowish, clamped, 3–8 μm wide, with cylindrical and blunt ends. Stipitipellis: composed of hyphae forming a trichoderm, cylindrical, thin-walled, yellowish, clamped, 4–6.5 μm wide, with cylindrical-to-subclaviform ends. Hyphae of the context: woven, cylindrical to swollen, thin-walled, hyaline with orange refringent drops, clamped, 5.5–15 (25) μm wide. Basal mycelium: white, composed of woven hyphae, cylindrical, thin-walled, clamped, 3–5.5 μm wide, with some ampullate septa (6–10.5 μm) Figure 2 and Figure 3.

### 3.4. Material Examined

FRANCE. Parc de Grignon (Seine-et-Oise), sous des sapins d’origine américaine, October 1953, leg. Heim R., PC (authentic material of *H. heimii*). 

ITALY. Liguria, “Le Manie”, 30 December 2022, leg. Cartabia M., herb M. Cartabia 20221230-2. Tuscany, Livorno, Parco Archeologico P/B Baratti, under *Quercus ilex*, 20 November 2010, leg. Cecchini A. & Narducci R., PI 62035. Tuscany, Lucca, Cipressata di S. Agnese, 12 km E of Poggibonsi, on slope in forest dominated by *Cupressus sempervirens* with scattered *Quercus ilex* & *Pinus*, 5 November 1996, Vesterholt J., JV96-401 (C-F-28274). Tuscany, Lucca, Villa Grabau, 11 December 1993, leg. Romanini M., det. Gori L., Gori 1232 (AGMT 10413). Tuscany, Pisa, Forreste Demaniali del Berignone, Monte Saldano, under *Pinus*, 8 November 1996, JV96-481 (C-F-26889). Tuscany, Pisa, Riparbella, Ortocavoli, under *Quercus* with *Erica*, 26 December 2022, leg. Cristiano A., AGMT 11293. Tuscany, San Vincenzo, Rimigliano, under *Quercus ilex*, 28 November 2009, AGMT 1909. Tuscany, Santa Maria del Giudice, under *Quercus ilex* and *Cupressus*, 31 December 2015, leg. Cecchini A. & Narducci R., PI 62033; under *Quercus ilex,* 10 November 1994, PI 62034; 12 December 2007, PI 62035. 

SPAIN. Álava, Aiala, Beotegi, 30TVN9371, 350 m, under *Pinus*, 5 November 1988, leg. Salcedo I. & Grupo 111, BIO-Fungi 2462. Álava, Barrio, Valdegovía, 30TVN7923, 700 m, under *Quercus rotundifolia*, 12 November 2002, leg. Olariaga I., BIO-Fungi 9679; BIO-Fungi 9680; 9 December 2004, BIO-Fungi 10464; under *Pinus sylvestris* on calcareous soil, 8 November 2007, BIO-Fungi 12677. Álava, Valdegovía, Osma, 30TWN9548, 600 m, *Quercus rotundifolia* forest on rich ground, 17 October 2004, leg. Olariaga I., BIO-Fungi 10461. Álava, Zigoitia, Apodaka, 30TWN2152, 550 m, under evergreen *Quercus* on the ground, 31 October 1987, leg. Salcedo I. & Grupo 111, BIO-Fungi 928. Álava, Kanpezu, 30TWN6325, under *Quercus rotundifolia* on calcareous ground, 4 December 2008, leg. Sarrionandia E. & Olariaga I., BIO-Fungi 12900. Burgos, Río de Losa, Valle de Losa, 30TVN7655, 600 m, under *Pinus sylvestris* on rich ground, 10-Sep-2004, leg. Olariaga I., BIO-Fungi 10475. Barcelona, Sant Celoni, Olzinelles, 31TDG6012, 300 m, *Quercus suber* on acidic soil, 14 October 2005, leg. Olariaga I. & Felipe A., BIO-Fungi 11128. Bizkaia, Sukarrieta, Txatxarramendi, 30TWP2404, 10 m, under *Quercus ilex* on calcareous soil, 17 October 2008, leg. Olariaga I., BIO-Fungi 12898. Córdoba, Priego de Córdoba, Peñasdoblas-Hortezuel, 30SUG9638, 900 m, en prado con *Quercus ilex* subsp. *ballota*, en suelo, 19 December 2002, leg. Gómez J., Pulido E. & Moreno B., JA 652. Castellón, Querol, 30SVH4859, 850 m, 5 December 1993, MA-Fungi 44420. Cuenca, Alcalá de la Vega, El Cubillo, proximidades de la Fuente de el Rebollo, 30TXK3034, 1300 m, under *Pinus nigra* ssp. *salzmannii*, 5 October 1997, leg. Daniëls P.P., MA-Fungi 37567. Gipuzkoa, Zumaia, Zuloaga-Artadi, 30TWN6194, 25 m, under *Quercus ilex*, 4 December 2004, leg. Albizu J.L., ARAN-Fungi A3083193. Girona, Sant Salvador, near Olot, NE of Gerona, in calcareous forest with *Quercus ilex*, 25 October 2005, leg. Vesterholt J., JV05-767 (C-F-44182). Granada, Huetor-Santullán, Arroyo de Fardes-Fuente de los Potros, 30SVG5929, 480 m, en bosque con *Quercus rotundifolia*, en suelo, 9 December 2002, leg. Capilla A., JA 3075. Huelva, Alrededores de Almonasterio la Real, alcornocal, 23 November 1991, leg. Ortega A., Esteve-Raventós F. & Moreno G., GDAC 37999. Huesca, Anso, 30TXN8033, 900 m, under *Pinus sylvestris*, 22 October 2002, leg. Ibarguren M.I. & Olariga I., BIO-Fungi 9684. Huesca, Bernués, Jaca, 30TXN9806, 1000 m, under *Quercus rotundifolia*, 21 October 2003, leg. I. Olariaga, BIO-Fungi 9973. Huesca, Banastás, under *Quercus rotundifolia* on calcareous soil, 12 December 2018, leg. García J. & Ballester L., LB18121204. Jaén, Cambil, Mata Bejid, 30SVG5574, 1150 m, en bosque con *Quercus rotundifolia*, en suelo, 6 November 2002, leg. Capilla A., JA 3132. Jaén, Los Pitillos, Embalse Quebrajero, encinar, terrícola, 2 November 1990, leg. A. Ortega, GDAC 34141. Jaén, mata Bejid, encinar, 7 February 1980, GDAC 31480; encinar, 27-11-1990, GDAC 36570. La Rioja, Torrecilla en Cameros, under *Pinus sylvestris*, 20 December 2014, leg. Ballester L. & Grupo Micológico Verpa, LB14122007. Mallorca, Near Cala Mayor, 14 January 1979, leg. Rabenborg P., det. Maas Geesteranus M.A., C-F-157313. Mallorca, near Valldemosa, in mixed forest, 17 January 1979, leg. Rabenborg P., det. Maas Geesteranus M.A., C-F-157314. Mallorca, Sa Communa de Bunyola, 31SDD7695, 620 m, under *Quercus ilex* on rich ground, 21 December 2006, BIO-Fungi 11726. Zaragoza, Barranco de la Hoya del Almendro, Vera del Moncayo, 30TXM0629, 700 m, under *Quercus rotundifolia*, 21 October 2003, leg. Olariaga I., BIO-Fungi 9990. 

SWEDEN. Gotland, Lickershamn, Jungfruklint, 23 September 2009, under *Pinus sylvestris* and *Picea abies* on calcareous ground, K. Hansen & I. Olariaga, S F-156583.

SWITZERLAND. Valais, Sierre, under *Pinus sylvestris* on calcareous soil, 30 August 2007, Felipe A. & Olariaga I., BIO-Fungi 12687; BIO-Fungi 12361. 

## 4. Discussion

The predominant white colour and the small spores support *Hydnum albidum* s. auct. pl. eur. to be accommodated in *Hydnum* subg. *Alba* Niskanen & Liimat. [5]. Within this group, *H. subcremeoalbum* Tedersoo, Liimat. & Niskanen, *H. zongolicense* Garibay-Orijel and *H. treui* Tedersoo, Liimat. & Niskanen morphologically differ by their more rounded, globose-to-subglobose spores (Qm < 1.1) [5]. The rest of the species assigned to *Hydnum* subg. *Alba*, *H. albidum* Peck, *H. alboaurantiacum* Swenie & Matheny and *H. creomeoalbum* Liimat. & Niskanen have similar spores compared to *H. albidum* s. auct. pl. eur., but none of them occur in Europe, and the available molecular data suggest that they are not conspecific with it. Interestingly, according to the extensive material studied, *H. albidum* s. auct. pl. eur. possesses a characteristic never mentioned for any other *Hydnum* species, i.e., the presence of rounded, < 1 µm crystals on the hyphae of spine apices [18] (Figure 142c). Nevertheless, more detailed studies on other species of *Hydnum* subg. *Alba* are needed to determine whether this characteristic is restricted to *H. albidum* s. auct. pl. eur. or has been overlooked in other species. All the European material of *Hydnum albidum* s. auct. pl. is morphologically homogeneous, and available ITS sequences show little divergence, as described above. It has been recorded in association with coniferous or broadleaf ectomycorrhizal hosts [18], and possibly with *Helianthemum* [17,20], showing a strong preference for calcareous ground. Although red-listed in some countries, such as Norway [41] and Sweden [42], *H. albidum* s. auct. eur. pl. is a common and widespread species in Mediterranean Europe, especially under evergreen oaks (*Quercus ilex*, *Q. rotundifolia*) on calcareous soils [18].

### 4.1. Study of the Name Hydnum Pallidum Raddi

*Hydnum pallidum* Raddi was validly published as a description was provided [21] (p. 353). *Hydnum pallidum* was described from Boboli Gardens (Florenze, Tuscany) as an entirely white-pale *Hydnum* with numerous spines. The drawing provided by Raddi (Table 13, Figure 8; erroneously referred to as “Tav.: V. Figure 8”) also shows an entirely white, small *Hydnum* that conforms beyond doubt to *H. albidum* s. auct. pl. eur. The only other European species that produces nearly completely white basidiomata, *H. boreorepandum*, is a boreal species [17], and hence its occurrence in the Florence area, characterized by a Mediterranean climate, (Csa type according to Köppen–Geiger’s classification [43], is difficult to conceive. Unfortunately, no original specimen of *H. pallidum* exists at FI and PI, where most of Raddi´s material is kept.

A more thorough search for information on *H. albidum* s. auct. pl. eur. shows that it is a widespread edible mushroom in Italy, even included in regional lists of commercialized species [44] and popularly called “Steccherino bianco” [45]. A more specific search in Tuscany revealed several bibliographic records [46,47,48] and treatments in popular websites of mushrooms [49,50]. Furthermore, we examined seven herbarium specimens (see material examined) from different sites in Tuscany. The morphological examination of those specimens and the sequence data obtained have confirmed that they belong to *H. albidum* s. auct. pl. All the information gathered shows that *H. albidum* s. auct. pl. is a widespread and rather common species in Tuscany and fully supports that the species described by Raddi as *H. pallidum* is conspecific with *H. albidum* s. auct. eur. In order to be able to check molecular characters and to support our interpretation, we propose here an epitype collected in Tuscany for *H. pallidum*.

### 4.2. Study of the Name Hydnum Album Fr.

The earlier publication of *Hydnum album* by Raddi [21] (p. 361), not validly published because it is lacking a description (see under excluded names further down), does not affect the legitimacy of *H. album* Fr. [22]. Although Fries included it among the “Hydnorum stipitatorum species mihi dubia” (doubtful species), *H. album* Fr. was validly published under Art. 36.1 [51]. Under *H. album* Fr., Fries [22] included the illustration provided by Micheli [37] for his “Erinaceus, esculentus, albus, crassus”, which is represented in Tab 72, Figure 2 (erroneously referred to as “Table 71. Figure 2” by Micheli). The latter author described a white edible mushroom called “Steccherino” or “Dentino bianco”, which occurred in several habitats near Florence in October and was sold in the markets at that season. Micheli’s illustration shows a hydnoid stipitate basidioma that, considering the white colour mentioned by Micheli, conforms to *H. albidum* s. auct. eur. In conclusion, there is little doubt that the fungus described by Micheli [37] (p. 132) is what has been called *Hydnum albidum* by European authors. 

Some confusion has existed as to the attribution of the name *H. album* to Fries or Persoon. As Maas Geesteranus [6] (p. 140) noted, Persoon did not refer to Fries [52] (p. 249) or Persoon [53], even though Persoon himself cited the same Micheli plate [37] (Table 72, Figure 2) as Fries under his treatment of *H. album*. Probably due to the fact that Persoon provided a more detailed opinion on the interpretation of *H. album*, Maas Geesteranus ascribed *H. album* to Persoon. Although Persoon did not link his *H. album* with Fries, both authors included a single element eligible as a type under their treatments, i.e., the Micheli plate cited above. *Hydnum album* Pers. cannot be considered a later homonym of *H. album* Fr., as both names would be based on the same type, and Art. 53 cannot be applied [51]. Thus, we consider Persoon's *H. album* as a later treatment of *H. album* Fr. that does not merit recognition as a different name.

### 4.3. Study of the Name Hydnum Heimii Maas Geest

*Hydnum heimii* was proposed as a new name for *Sarcodon abietinus* R. Heim, invalidly published by Heim [38] as no Latin diagnosis was provided (Art. 39.1 [51]). The diagnostic features noted by Heim [38,39] were the overall pale colour (cream white) and the yellowish-olivaceous pileus centre. Remarkably, Heim [38,39] described the spores as 6–7 × 4.8–5 µm. Maas Geesteranus [6] failed to obtain any of the specimens cited by Heim [38] on loan, but was able to check a specimen collected in the type locality in 1953. During the visit by I. Olariaga to the PC herbarium, the type of *H. heimii* could not be located either, but he was able to examine the specimen collected in 1953, regrettably in a rather poor condition. The specimen contains around 10 dry basidiomata of a dark reddish brown colour. The spore (5.5–7 × 3.5–4.5(5) µm) measurements are very similar to those provided by Heim, and we observed basidia to be 5–6 µm broad. Both the spore and basidia sizes fall within the range of *H. albidum* s. auct. pl. eur. Considering the cream–white basidioma colour described for *H. heimii* [38,39] and the microscopic characters mentioned, we conclude that *H. heimii* is conspecific with what European authors have called *H. albidum*. The remaining known species in Europe possess larger spores (e.g., 7.1–9.2 × 5.9–8.7 µm in *H. repandum*, Olariaga [3]) and broader basidia (e.g., 8–11 µm in *H. repandum* [3]) in our experience. The fact that Maas Geesteranus [40] treated *H. heimii* as a synonym of *H. repandum* can be explained by the fact that he included within *H. repandum* material from several continents that is known to belong to several species today. He therefore treated *H. repandum* as an extremely variable species and saw a continuum in many characters such as in the basidioma size, colour and spore size in his treatment in 1975. 

### 4.4. Excluded, Not Validly Published or Illegitimate Names That Refer to White-Coloured Hydnum

*Hydnum album* Raddi, Mem. Mat. Fis. Soc. Ital. Sci. Modena, Pt. Mem. Fis. 13(2): 361. 1807. Type: No type specimen exists in the Raddi herbarium (PI, FI).

Raddi [21] (p. 361) listed this name as “*Hydnum album* Nob.”, but did not provide any description for it. Therefore, it is regarded here as a nomen nudum, as Maas Geesteranus [54] regarded it, and therefore invalidly published.

*Hydnum candidum* J.C. Schmidt in Kunze & Schmidt, Mykol. Hefte 1: 89. 1817, nom. sanct. non Hydnum candidum Willd., Bot. Mag. (Römer & Usteri) 2: 14. 1788. ≡ *Sarcodon candidum* (J.C. Schmidt) Quél., Enchir. fung.: 189. 1886. Type: No original material exists. 

The gelatinous pileus, the hyaline spines and the context that turns purple do not fit any known species of *Hydnum,* as circumscribed today. *Hydnum candidum* is therefore excluded from *Hydnum* here.

*Hydnum medium* Pers., Observ. mycol. 2: 97. 1800. [1799]. ≡ *Hydnum repandum* [unranked] denudatum [unranked] *albidum* Fr., Obs. Mycol. 1: 139. 1815. Type: No original specimen exists in L nor any original illustration.

Persoon [55] described *H. medium* as having a glabrous and pallid pileus, acute thin spines and a short white stipe. He further argued that the aculei and the pileus surface were close to *H. repandum*, whereas the colour of the spines conformed to *H. rufescens*. The normal size and the pallid pileus—not described as white—suggest this name is most likely to be a synonym of *H. repandum*, as considered by Maas Geesteranus [6] (p.136), under *H. repandum* var. *repandum*, but the possibility of it belonging to *H. pallidum* cannot be ruled out. Unfortunately, no type specimen appears to exist. With the information available up to date, we consider *H. medium* a doubtful name.

*Hydnum repandum* var. *albidum* Cejp, Bull. Internat Acad. Sci. Bohême 31: 82. 1928 (nom. illeg., Art. 53.3, later homonym of *Hydnum repandum* var. *albidum* (Peck) Bres). Type: Czech Republic, distr. Ricany u Prahy, in pineto apud pag. Hrusice, IX 1925, leg. Velenovsky (PRC).

The spore measurements provided by Cejp [56] were 7–7.5 × 6–8 µm and therefore do not conform to those of *H. pallidum*.

## 5. Conclusions

A comprehensive nomenclatural revision revealed the existence of three earlier names for the species that has been traditionally called *H. albidum* in Europe and for which the name *H. reginae* was coined recently. The adoption of the name *H. pallidum* for *H. albidum* s. auct. pl. eur. will bring nomenclatural stability as it is unlikely that any earlier synonym of it exists. We hope this study encourages the in-depth study of other *Hydnum* names described before the advent of the molecular era, among which further earlier synonyms of the later described species might be found.

## Figures and Tables

**Table 1:** Sequenced specimens used for the phylogenetic analysis of this study, with GenBank or UNITE (UDB) accession numbers for ITS and LSU regions. Newly generated sequences in this study are in bold, and sequences from type specimens are indicated. Original identifications of the sequences are provided between parentheses.

				GenBank/UNITE No.	
Species ^1^	Voucher ^2^	Type	Country ^3^	ITS	LSU
*Hydnum albidum*	CORT012029	Epitype	USA	NR164025	—
*Hydnum albidum*	AH179(NBM)		Canada	OQ235288	OQ235288
*Hydnum alboaurantiacum*	MH16004		USA	MH379904	—
*Hydnum alboluteum*	TUMH63988	Holotype	Japan	NR176695	—
*Hydnum albomagnum*	TENN073062	Epitype	USA	NR164031	—
*Hydnum albopallidum*	TUMH63997	Holotype	Japan	NR176696	LC717904
*Hydnum brevispinum*	IFP019464	Holotype	China	NR175734	MW979559
*Hydnum boreorepandum*	TUMH64006		Japan	LC621815	LC717881
*Hydnum cremeoalbum*	TUMH64024		Japan	LC621823	LC621823
*Hydnum ellipsosporum*	FD3281		Switz	KX086215	KX086217
*Hydnum flabellatum*	IFP 019459	Holotype	China	NR175732	MW979556
*Hydnum flavidocanum*	IFP 019460	Holotype	China	NR175727	MW979545
*Hydnum ibericum*	LUGOECC16112305		Spain	MW376665	—
*Hydnum itachiharitake*	TUMH64033	Holotype	Japan	LC621830	LC621830
*Hydnum longibasidiatum*	IFP019462	Holotype	China	NR175726	MW979541
*Hydnum melleopallidum*	SMI356	Holotype	Canada	FJ845406	FJ845406
*Hydnum minus*	TUMH64050		Japan	LC621842	LC717910
*Hydnum neorepandum*	MO313671		USA	MH156048	MH156048
*Hydnum olympicum*	MO313677		USA	MH156204	MH156204
*Hydnum oregonense*	MO313676		USA	MH158252	MH158252
*Hydnum orientalbidum*	TUMH 64056		Japan	LC621855	LC621855
*Hydnum orientalbidum*	TUMH 62998	Holotype	Japan	—	LC717908
*Hydnum pallidocroceum*	IFP019466	Holotype	China	NR175731	MW979554
*Hydnum pallidomarginatum*	IFP019468	Holotype	China	NR175730	MW979552
*Hydnum pallidum (H.a.)*	MA-Fungi 40149		Spain	AJ534975	—
*Hydnum pallidum (H.a.)*	LB14122007		Spain	**OR824947**	—
*Hydnum pallidum (H.a.)*	JV96-481 (C)		Italy (T)	**OR824944**	—
*Hydnum pallidum (H.a.)*	JV05-767 (C)		Spain	**OR824945**	**OR821820**
*Hydnum pallidum (H.a.)*	MC20221230_2		Italy	**OR824949**	—
*Hydnum pallidum (H.a.)*	C-F-157314		Spain	**OR824946**	—
*Hydnum pallidum (H.a.)*	AGMT 11293	Epitype	Italy (T)	**OR824948**	**OR821821**
*Hydnum pallidum (H.r.)*	K-M 265258	Holotype	UK	ON502618	ON502618
*Hydnum pallidum (H.r.)*	TUF106235		Estonia	UDB011441	UDB011441
*Hydnum pallidum (H.r.)*	TUF101782		Sweden	UDB016627	UDB016627
*Hydnum pallidum (H.a.)*	LJU-GIS 1341		Slovenia	AJ534974	AJ534974
*Hydnum pinicola*	TUMH 64004	Holotype	Japan	NR176697	—
*Hydnum repando-orientale*	TUMH 64069		Japan	LC621873	LC717903
*Hydnum repandum*	CNV70		USA	MT345247	MT345247
*Hydnum rufescens*	UP504		Sweden	OL739363	OL739363
*Hydnum sphaericum*	IFP 019470	Holotype	China	NR175729	MW979549
*Hydnum subalpinum*	TUMH64630		Japan	LC717915	LC717894
*Hydnum subcremeoalbum*	TUF110688		PNG	UDB013289	—
*Hydnum subtilior*	TENN 073034	Holotype	USA	NR164029	—
*Hydnum tenuistipitum*	IFP 019476	Holotype	China	NR175733	MW979557
*Hydnum tomaense*	TUMH64086	Holotype	Japan	NR176701	LC717907
*Hydnum treui*	TUF110403		PNG	UDB013043	—
*Hydnum umbilicatum*	OUC99371		Canada	DQ367903	DQ367903
*Hydnum vesterholtii*	HKAS92341		China	KU612562	KU612647
*Hydnum washingtonianum*	MO319176		USA	MH482744	MH482744
*Hydnum zongolicense*	GO-2010-142a	Holotype	Mexico	KC152121	—
*Sistotrema muscicola*	KHL 11721		Finland	AJ606040	AJ606040

^1^ *H.a.*: *Hydnum albidum*; *H.r.: Hydnum reginae.* ^2^ MO: Mushroom Observer. ^3^ Switz: Switzerland; Italy (T): Tuscany (Italy); UK: United Kingdom; PNG: Papua New Guinea.

## Data Availability

Publicly available datasets were analyzed in this study. These data can be found here: https://www.ncbi.nlm.nih.gov (accessed on 28 November 2022).
